# Infra-Slow Oscillation (ISO) of the Pupil Size of Urethane-Anaesthetised Rats

**DOI:** 10.1371/journal.pone.0062430

**Published:** 2013-04-26

**Authors:** Tomasz Blasiak, Artur Zawadzki, Marian Henryk Lewandowski

**Affiliations:** 1 Department of Neurophysiology and Chronobiology, Institute of Zoology, Jagiellonian University, Krakow, Poland; 2 Department of Automatics and Biomedical Engineering, AGH University of Science and Technology, Krakow, Poland; University of Alberta, Canada

## Abstract

Multiplicity of oscillatory phenomena in a range of infra-slow frequencies (<0.01 Hz) has been described in mammalian brains at different levels of organisation. The significance and manifestation in physiology and/or behaviour of many brain infra-slow oscillations (ISO) remain unknown. Examples of this phenomenon are two types of ISO observed in the brains of urethane-anaesthetised rats: infra-slow, rhythmic changes in the rate of action potential firing in a few nuclei of the subcortical visual system and a sleep-like cycle of activation/deactivation visible in the EEG signal. Because both of these rhythmic phenomena involve brain networks that can influence autonomic nervous system activity, we hypothesised that these two brain ISOs can be reflected by rhythmic changes of pupil size. Thus, in the present study, we used simultaneous pupillography and ECoG recording to verify the hypothesised existence of infra-slow oscillations in the pupil size of urethane-anaesthetised rats. The obtained results showed rhythmic changes in the size of the pupils and rhythmic eyeball movements in urethane-anaesthetised rats. The observed rhythms were characterised by two different dominant components in a range of infra-slow frequencies. First, the long component had a period of ≈29 minutes and was present in both the irises and the eyeball movements. Second, the short component had a period of ≈2 minutes and was observed only in the rhythmic constrictions and dilations of the pupils. Both ISOs were simultaneously present in both eyes, and they were synchronised between the left and right eye. The long ISO component was synchronised with the cyclic alternations of the brain state, as revealed by rhythmic changes in the pattern of the ECoG signal. Based on the obtained results, we propose a model of interference of ISO present in different brain systems involved in the control of pupil size.

## Introduction

Brain infra-slow oscillations (ISO; frequency <0.1 Hz) were described for the first time by Aladjalova [1] at the level of local field potential recorded in the rabbit neocortex. Since then, using the diverse recording techniques available, the ISO phenomenon has been documented in various structures of animal brains (for review see [2]). Infra-slow oscillations were observed in the variation of membrane potential of single neurons *in vitro* [3], in the changes in neuronal firing rate or local field potentials *in vivo* [4]–[11], and in the activity of large neuronal networks reflected by full band EEG and/or fMRI signals from human and animal brains [12]–[19]. These studies revealed that some types of ISO participate in patterning the occurrence of higher frequency oscillations, both physiological (e.g., alpha rhythm; [15], [20]) and pathological (e.g., epileptic seizures; [18]), or can influence human psychophysical performance [17]. The occurrence of ISO in fMRI BOLD signals from the resting human brain has been recently used to define different resting state networks (RSN; [12], [13], [15]).

Despite an increasing number of research studies describing various infra-slow oscillations in different regions of animal and human brains, little is known about the significance and manifestation of these oscillations in physiology and/or behaviour. An example of this phenomenon is the well-documented ISO observed in the brains of urethane-anaesthetised rats. We have shown that in this preparation, some neuronal populations in the intergeniculate leaflet (IGL; [8], [21]) and olivary pretectal nucleus (OPN; [11]) display stable infra-slow, rhythmic changes in the rate of action potential firing. Another infra-slow rhythm present in the brains of urethane-anaesthetised rats is a sleep-like cycle of activation/deactivation clearly visible in the EEG signal [22]. We hypothesise that these two brain ISOs can be reflected by pupil size changes in urethane-anaesthetised animals. Two major facts support this idea. First, the olivary pretectal nuclei are the key elements of the pupillary light reflex, directly exciting the parasympathetic branch of the autonomic nervous system (ANS), which innervates the sphincter pupillae and, thus, constricts the pupil (i.e., miosis). Second, the sleep-like cyclic structure of EEG activation/deactivation observed during urethane anaesthesia depends upon muscarinic neuromodulation [22], (for review see [23]) and it has been shown that stimulation of central muscarinic receptors induces pupil dilation via the cervical sympathetic nerves [24].

In fact, it has been shown that under constant accommodation and lighting, the human pupil slowly oscillates [25] and that these changes increase in amplitude as the individual becomes drowsy or sleepy [26], [27]. It has been suggested that these sleepiness-related pupillary oscillations may result from changes in the balance between the activity of the sympathetic and parasympathetic branches of the ANS [27]. Nevertheless, the neuronal mechanism of this phenomenon remains largely unknown, partly because of the lack of a convenient animal model with which to study the link between the brain ISO and pupil size fluctuations. Thus, in the present study, we used simultaneous pupillography and EEG recording to verify the hypothesised existence of infra-slow oscillations in the pupil size of urethane-anaesthetised rats.

## Materials and Methods

The experiments were approved by the Ethical Committee of Jagiellonian University and were performed in accordance with institutional guidelines for the care and use of experimental animals established by the European Communities Council Directive of 24 November 1986 (86/609/EEC). The protocol was approved by the Committee on the Ethics of Animal Experiments of the Jagiellonian University (Permit Number: ZI/447/2007). All efforts were made to minimise animal suffering and reduce the number of animals used.

### Surgical Procedures

Experiments were conducted on 13 adult male Wistar rats (250–350 g). The animals were housed and bred in standard conditions in a colony at the Institute of Zoology, Jagiellonian University. The animals were maintained under a 12∶12 light–dark cycle (lights on at 8 a.m. local time) with water and food available *ad libitum*. All surgical procedures were conducted under deep anaesthesia induced by an i.p. injection (1 h after lights onset) of urethane solution (1.5 g urethane/1000 g body weight; diluted in saline). Throughout the operation, electrocorticogram (ECoG) and electrocardiogram (ECG) signals were monitored, and the core body temperature was maintained at 37°C by an automatic heating pad. At no stage of the recordings was the application of additional doses of anaesthetic needed. ASI Instruments Inc. (Warren, MI, USA) stereotaxic equipment was used for mechanical fixation of the animals’ heads. The equipment consisted of a small animal stereotaxic frame (SF-1450AP) equipped with a rodent adapter for adult rats (RA-100) and standard ear bars (18° tip; EB-918). Rats were carefully mounted on the ear and incisor bars to assure correct and stable positioning of the head relative to the stereotaxic frame. A sagittal incision on the top of the head was performed, and the skin and soft tissues covering the bone were retracted laterally so that the bone sutures (coronal, sagittal, lambdoid) and temporal ridges were exposed and clearly visible. After the bregma point was determined as described [28], a craniotomy was performed to allow epidural implantation of a silver ball electrode for ECoG recording. The recording electrode was implanted in the right hemisphere over the primary visual cortex (VCx; area 17), with co-ordinates AP = −7.00 and ML = +4.00 [29]. The left and right eyelids were retracted and stabilised by surgical micro clips. A small drop of paraffin oil (Sigma, Poland; cat.no.: 76235) was applied to the exposed front of each eyeball to protect the corneas from drying out.

### Recording of Video and ECoG Signals

The ECoG signal was amplified using a CyberAmp 380 amplifier equipped with a preamplifier A-401 (Molecular Devices, CA, USA; final amplification: 2000×; filtration: 0.1–200 Hz) and digitised at the rate of 2 kHz. ECoG signal and time-synchronisation pulses (see below) were recorded using the CED micro1401 interface and Spike2 software (Cambridge Electronic Design, UK). Video of each eyeball was recorded by two identical video recording systems, each comprised of a stereomicroscope (PZO, Poland; model: MI 24U/S ENDO) equipped with a coaxial cold, halogen light source illuminator and video recording system (BOB OpticFibre Systems, Poland; model: BOB OM 100×2 equipped with colour CCD camera model: BOB-KE). The constant illumination level measured at the level of each eye was 10 000 lux (TES, Taiwan; model: 1336A). Each stereomicroscope was positioned in front of the corresponding eyeball, and the optic axes of the microscope and the eye were aligned during the ECoG deactivation phase. Video of each eye was recorded at a rate of 10 fps and with a resolution of 1024×768 pixels. The video signal was recorded over at least a full cycle of cortical activation/deactivation. A micro LED (Kingbright, Germany; model: KP-1608SURCK) emitting red light (650±28 nm) had been placed in the temporal corner of each eye in the field of vision of the video recording system. To time synchronise ECoG and video signals, the pulses (1.8 V, 100 ms, 0.01 Hz) generated by a programmable pulse generator (A.M.P.I, Israel; model: Master-8) were fed into micro LEDs and a digital input of micro1401 interface.

### Data Processing and Analysis

The video and electrophysiological signals were cropped and aligned according to time-synchronising pulses. The video signal was down sampled to 1 Hz, and each frame was analysed by a custom-written MatLab script (MathWorks, MA, USA) to calculate the area and centre of the ellipse fitted to the detected edges of the pupil. The spectral content of the ECoG signal was determined using Fast Fourier Analysis performed for consecutive 20-s-long epochs of the signal. Spectral analysis of the pupil size changes was performed using Fast Fourier Analysis applied to the whole video recording time. The cross-correlation between the values of the left and right eye pupil areas was sequentially calculated for a window of 300-s width shifting in 10-s steps over the data. Maximal and minimal values of the rhythmically changing pupil size were detected, and curves were mathematically fitted to the obtained values using MatLab software (MathWorks, MA, USA). The quantitative analyses are expressed as the mean ± S.E.M. Student’s t-test for dependent samples was used for statistical testing, and differences were considered significant at p<0.01. Statistical tests and calculations were performed using Statistica software (StatSoft, OK, USA).

## Results

**Figure 1:**
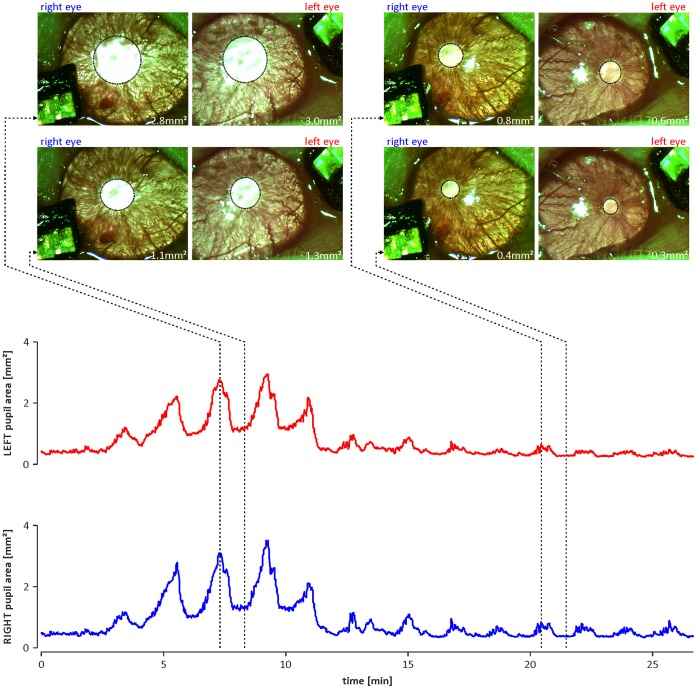
An example of oscillations in the size of the pupils of a urethane-anaesthetised rat. Top: frames of the left and right eyes taken at different recording times. Dotted ellipses indicate detected edges of the pupils, and the corresponding area of the pupil is given in the bottom right corner of each frame. Bottom: graphs showing the oscillatory changes of the areas of pupils observed simultaneously in the left (red line) and right (blue line) eyes. Both graphs have the same time scale. Dotted lines with arrows indicate the exact times at which the frames shown in the top were taken.

In 13 animals, simultaneous, bilateral video recording of the pupils and ECoG recording was performed under constant light conditions. In all animals, the pupil area of both eyes displayed clear oscillations (Fig. 1).

**Figure 2:**
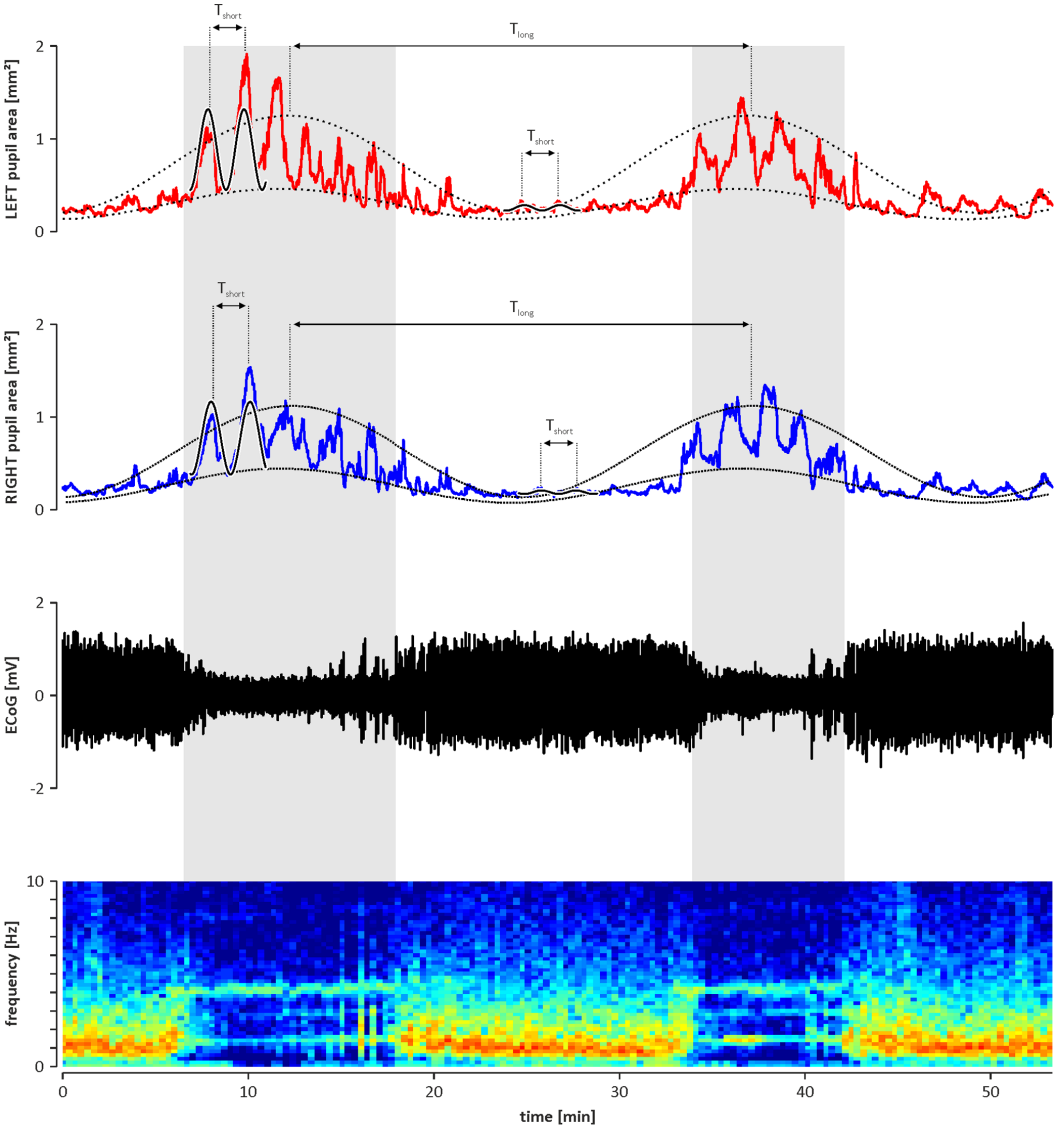
An example showing the two infra-slow components of oscillatory changes of the pupil size and its coincidence with cyclic alternations of brain state. Top: two diagrams showing the rhythmic changes simultaneously observed in the left (red line) and right (blue line) eyes. The long infra-slow component is indicated on both diagrams by the dotted curves that were mathematically fitted to the maximal and minimal values of the pupil area. The period (T_ISO_long_) of the long ISO is indicated on both diagrams. The short infra-slow component is indicated on both diagrams by solid lines that were mathematically fitted to the fragments of pupil size oscillations observed during ECoG activation and deactivation (see below). The period (T_ISO_short_) of the short ISO is indicated on both diagrams. Bottom: two diagrams showing cyclic alternations of the brain state. The black diagram shows the raw ECoG signal, and the colour surface plot below indicates the dominant frequency in the ECoG signal. The hottest (red) and coldest (blue) colours represent the highest and lowest values in the power spectrum at the particular frequency calculated for the window sliding over the raw ECoG signal (step 10 s; width 300 s). Activation phases, indicated by shaded areas, are characterised by dominant theta frequency (≈4 Hz) in the ECoG signal, and deactivation phases are characterised by dominant delta frequency (≈1 Hz) in the ECoG.

**Figure 3:**
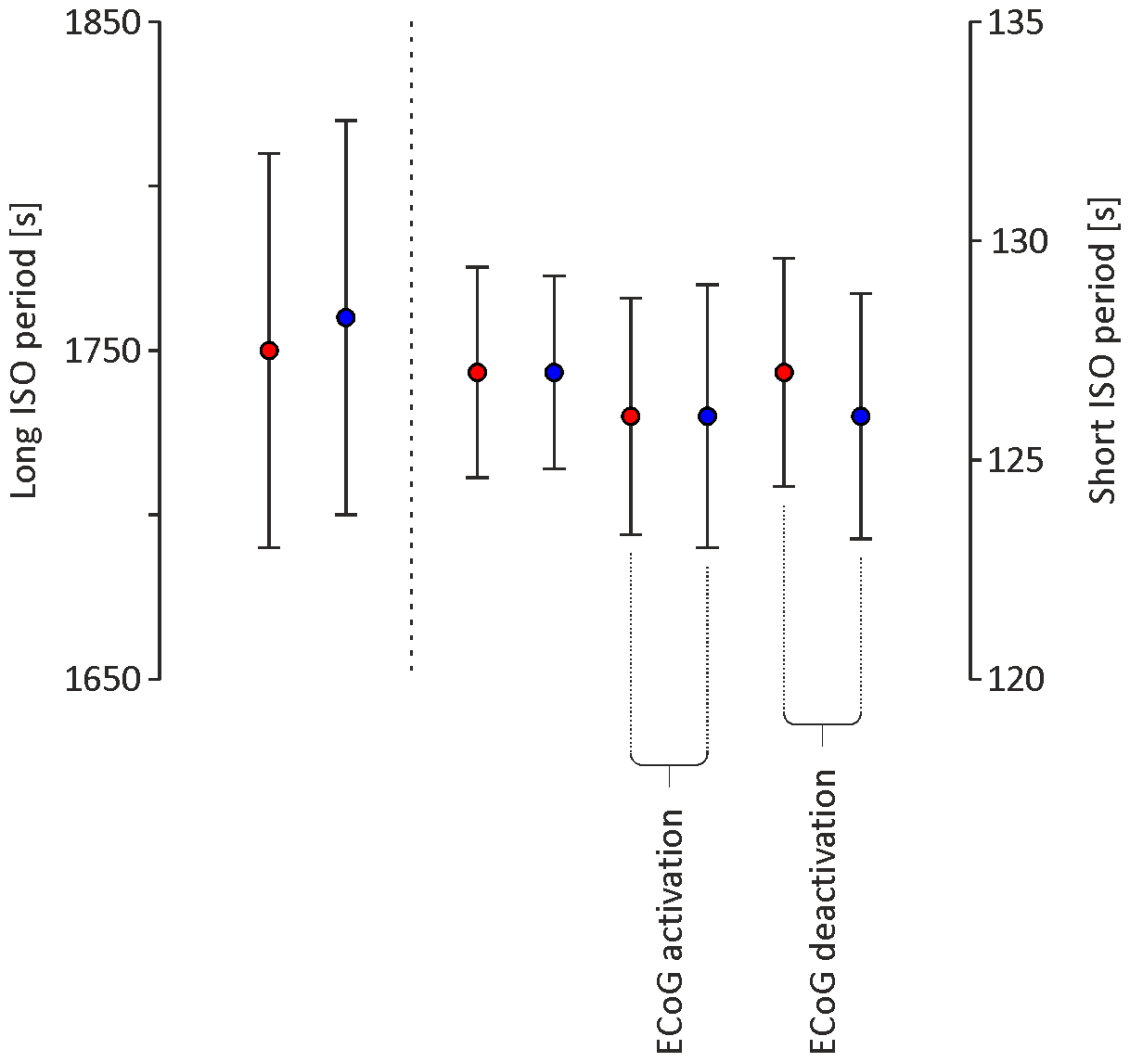
Graph showing the average period ± SEM (n = 13 for each value; ○) for the long ISO and short ISO components of oscillations observed in the size of the pupil of the left (red filled circles) and right (blue filled circles) eyes. The ECoG phase (i.e., deactivation and activation) is indicated by captions at the bottom of the graph if the value was determined for this phase only. Note the lack of statistically significant differences (p = 0.73; Student’s t-tests for paired data) between the values determined for long ISO and between the values determined for short ISO.

Spectral analysis of cyclic changes of the pupil area revealed two dominant infra-slow component oscillations in both eyes (i.e., frequency <0.1 Hz). The first component, subsequently called long ISO, was characterised by a mean period (T_long_) of 1750±60 s (n = 13; Fig. 2 and Fig. 3) for the left eye and 1760±60 s (n = 13; Fig. 2 and Fig. 3) for the right eye. The value of T_long_ did not differ significantly between the left and right eye (n = 13, p = 0.34; Student’s t-test for paired data; Fig. 3). The second component of infra-slow oscillation, subsequently called short ISO, was characterised by a mean period (T_short_) of 127±2.4 s (f_short_ = 0,008±0.0002 Hz; n = 13; Fig. 2 and Fig. 3) for the left eye and 127±2.2 s (f_short_ = 0,008±0.0001 Hz; n = 13; Fig. 2 and Fig. 3) for the right eye. The value of T_short_ did not differ significantly between the left and right eye (n = 13, p = 0.50; Student’s t-test for paired data; Fig. 3).

**Figure 4:**
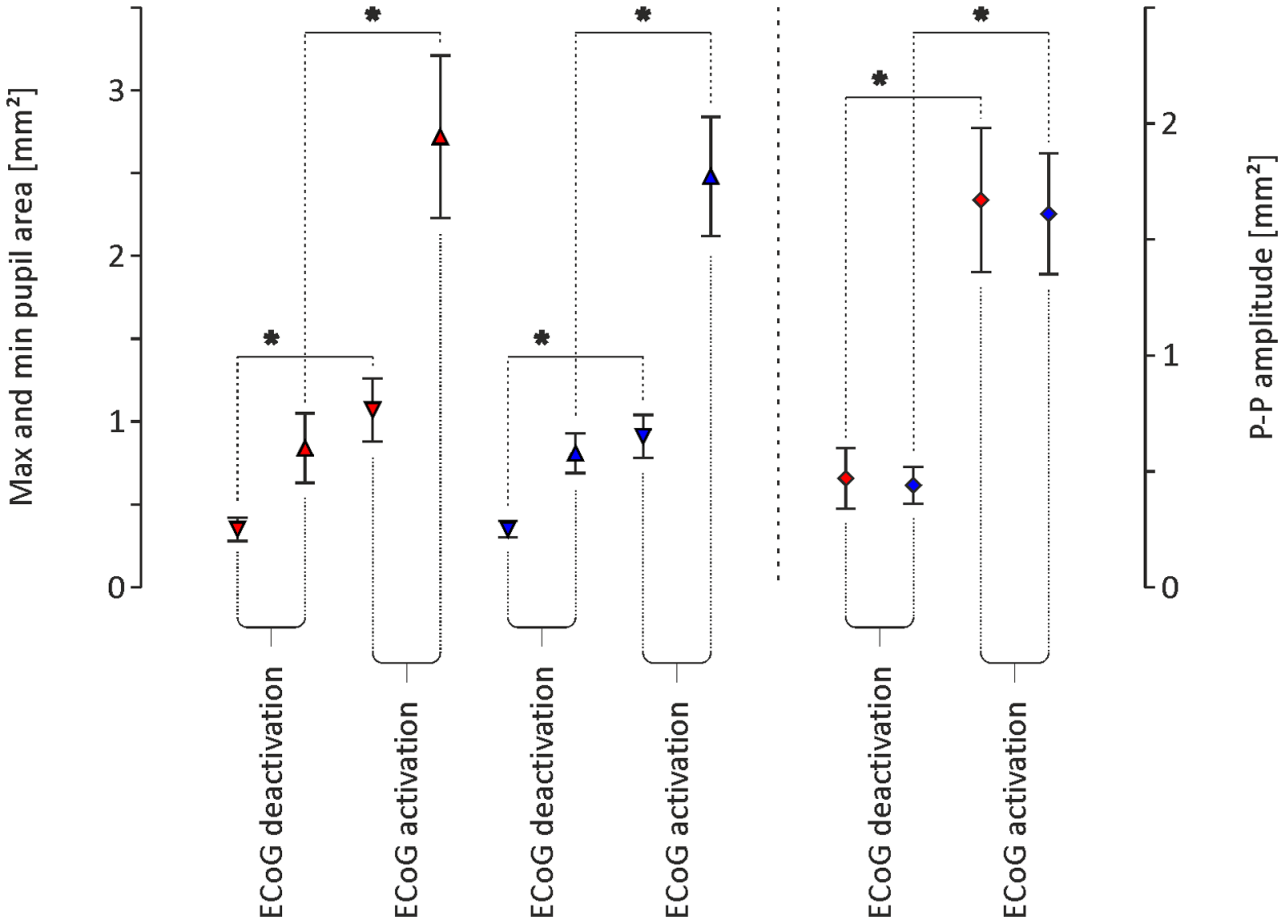
Graph showing the minimal (▿), maximal (▵) and peak-to-peak amplitude (⋄) average values ± SEM (n = 13 for each value) for infra-slow oscillations observed in the size of the pupil of the left (red filled symbols) and right (blue filled symbols) eyes. The ECoG phase (i.e., deactivation and activation) when the values were determined is indicated by captions at the bottom of the graph. Differences marked by asterisks are statistically significant (p<0.01; Student’s t-tests for paired data).

In all animals, the long infra-slow oscillation coincided with rhythmic alternation of the brain state between activated and deactivated ECoG patterns. During the activation phase (i.e., theta rhythm dominating in ECoG), the minimal and maximal sizes of the pupils were significantly higher than during the deactivation phase (i.e., delta rhythm dominating in ECoG; left eye pupil: min. 0.35±0.07 mm^2^ and max. 0.84±0.21 mm^2^ during deactivation vs. min. 1.07±0.19 mm^2^ and max. 2.72±0.49 mm^2^ during activation phase; right eye pupil: min. 0.35±0.05 mm^2^ and max. 0.81±0.12 mm^2^ during deactivation vs. min. 0.91±0.13 mm^2^ and max. 2.48±0.36 mm^2^ during activation phase; n = 13, p<<0.01; Student’s t-test for paired data; Fig. 2 and Fig. 4). Additionally, the amplitude of short infra-slow oscillation in the size of the pupils differed between the activation and deactivation phase observed in ECoG. During the activation phase, the amplitude of short ISO was significantly higher than during the deactivation phase (left eye pupil: 0.47±0.13 mm^2^ vs. 1.67±0.31 mm^2^, during deactivation vs. activation, respectively; right eye pupil: 0.44±0.08 mm^2^ vs. 1.61±0.26 mm^2^, during deactivation vs. activation, respectively; n = 13, p<<0.01; Student’s t-test for paired data; Fig. 4). There was no significant difference in the period of short ISO in the size of the pupils observed during deactivation and activation ECoG phases (left eye pupil: 126±3.0 s vs. 126±2.7 s; n = 13, p = 0.8; right eye pupil: 126±2.8 s and 127±2.6 s; n = 13, p = 0.7; Student’s t-tests for paired data; Fig. 3). Rhythmic alternations of the brain state between activated and deactivated ECoG patterns were unaffected by blockade of irises movements by bilateral, topical application of 1% atropine sulphate solution (Polfa, Poland; data not shown).

**Figure 5:**
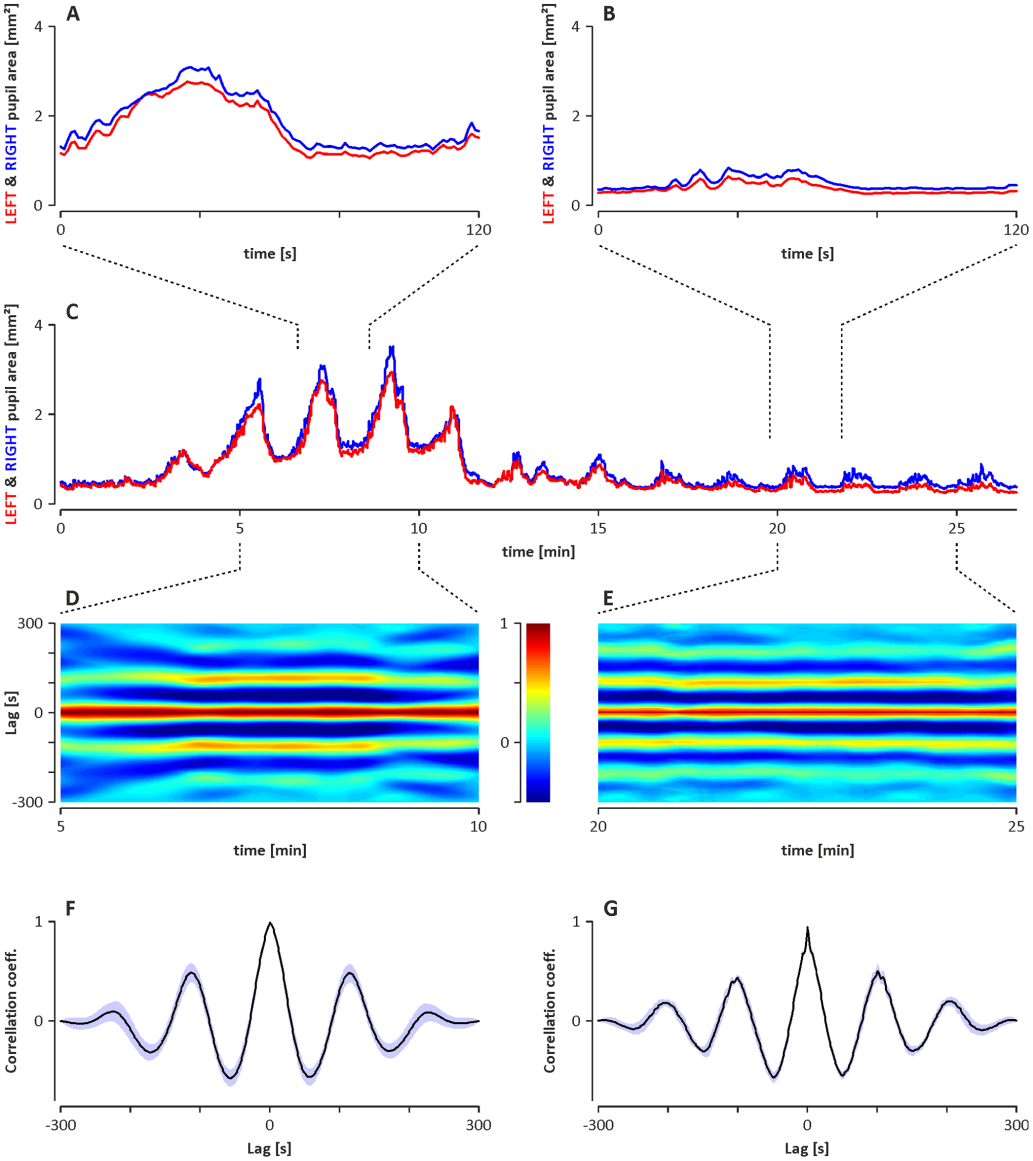
An example of the synchronisation of infra-slow oscillations of pupil areas observed simultaneously in both eyes. C: graph showing overlaid oscillations simultaneously observed in the pupil area of the left (red line) and right (blue line) eyes. A and B: graphs showing the magnified fragments of the recording shown in graph C. D and E: colour surface plots showing the cross-correlograms calculated with a sliding window (step, 10 s; width, 300 s) for two fragments of the recording shown in graph C. The y-axis denotes the time lag of the correlation function; the x-axis denotes the time range; and the cross-correlation value is coded by colour (colour scale is given on the inset). F and G: time-averaged cross-correlograms computed from D and E, respectively; the SDs of the calculated means are shown in violet. Note that the pupil area oscillations are synchronised between the eyes during ECoG activation (A, D, F) and deactivation (B, E, G).

In all animals, the rhythmic constrictions and dilations of the pupils underlying the observed infra-slow oscillations were synchronised between the left and right eye, as shown in the example in Figure 5.

**Figure 6:**
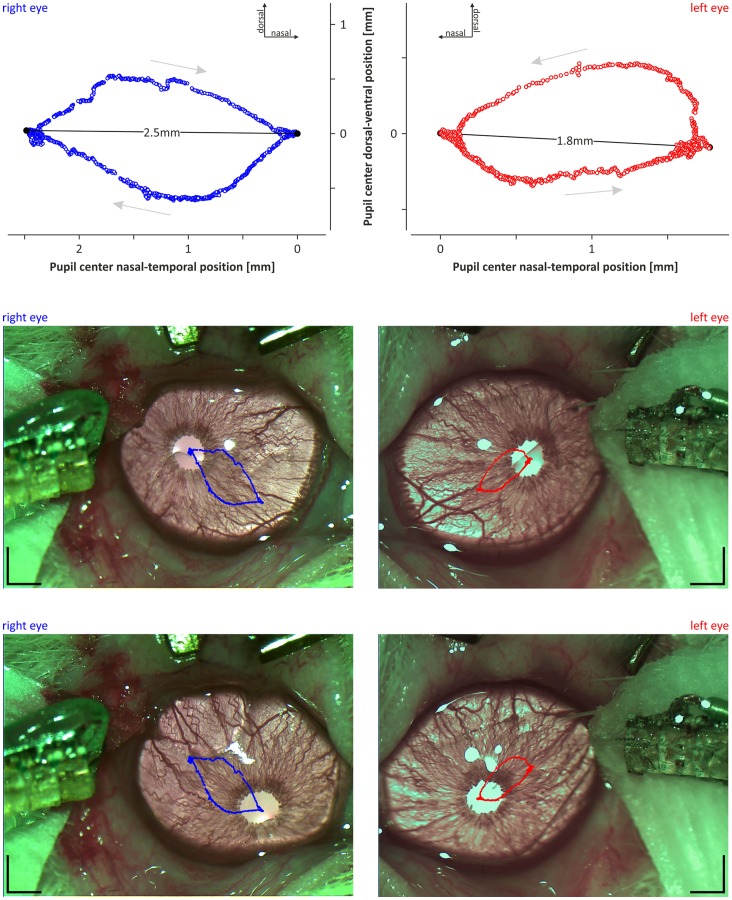
An example of the eyeball movements resulting in shifts in the positions of the pupil centres. Top: diagrams showing the shifts in the positions of the pupil centres of the left (red circles) and right (blue circles) eyes over the full ECoG activation-deactivation cycle. The zero of the nasal-temporal and dorsal-ventral axis was arbitrarily set at the most nasal position of the pupil centre. Grey arrows indicate the direction of the eyeball movements during cyclic transitions between ECoG deactivation (temporal positions) and activation (nasal positions). Solid lines with numbers indicate the distance between the most extreme pupil centre positions in the nasal-temporal direction. Bottom: frames of the left and right eyes taken when the pupil centre was located at the most temporal (upper images) and most nasal (lower images) positions. The graphs from the top of the figure have been overlaid over the frames to visualise the pathway of the pupil centres. The scale bar on all frames is 1 mm.

**Figure 7:**
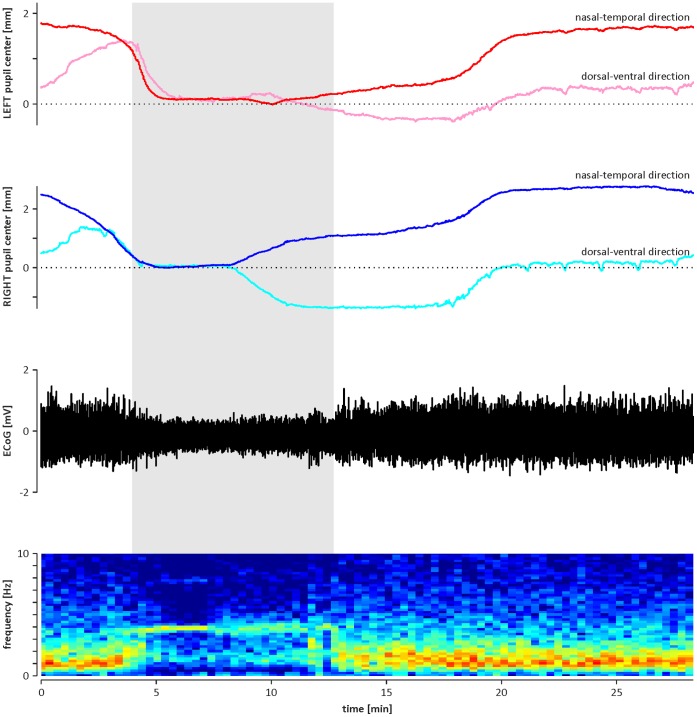
An illustration of a typical correlation of eyeball movements with brain-state changes. Top: diagrams showing the pupil centre movements in the nasal-temporal and dorsal-ventral directions, separately for the left (upper diagram) and right (lower diagram) eyes from the example in Figure 6. Bottom: two diagrams showing cyclic alternations of the brain state. The black diagram shows row ECoG signal, and the colour surface plot below indicates the dominant frequency in ECoG signal. The hottest (red) and coldest (blue) colours represent the highest and lowest values in the power spectrum at the particular frequency calculated for the window sliding over the raw ECoG signal (step 10 s; width 300 s). Activation phase, indicated by shaded area, is characterised by dominant theta frequency (≈4 Hz) in ECoG signal, and deactivation phase is characterised by dominant delta frequency (≈1 Hz) in ECoG. Note that the relocation of the pupil from the temporal to nasal position, concomitant with the transition from ECoG deactivation to activation, is markedly faster than the gradual shift of the pupil centre from the nasal to temporal position.

In all animals, concomitant with infra-slow oscillations in pupil size, recurring shifts in the position of the centre of the pupils were observed (Fig. 6). Both eyeballs were moving synchronously, i.e., one eyeball’s rotation in the nasal direction coincided with the nasal rotation of the contralateral eyeball. The same was true for other directions (temporal, dorsal and ventral; Fig. 6 and Fig. 7). The observed movements of both eyeballs were correlated with the cyclic pattern of activation and deactivation observed in the ECoG signal and were characterised by a mean period of 1730±50 s and 1750±50 s for the left and right eyes, respectively (n = 13, p = 0.4; Student’s t-tests for paired data). During the ECoG deactivation phase, the pupil centres were located in the most temporal position, and during the activation phase, the pupils were located in the most nasal position. The mean distance between the most nasal and temporal positions of the left pupil (2.1±0.2 mm) did not differ from the mean distance observed for the right pupil (2.0±0.2; n = 13, p = 0.7; Student’s t-tests for paired data). Relocation of the pupils from the temporal to nasal position was markedly faster than relocation of the pupils from the nasal to temporal position (Fig. 7).

## Discussion

### Summary of the Results

The results described in this paper have shown the presence of rhythmic changes in the size of the pupil and rhythmic eyeball movements in urethane-anaesthetised rats. Spectral analysis of the observed oscillatory phenomenon revealed two different, dominant components in recorded rhythms, both in a range of infra-slow frequencies (i.e., <0.1 Hz). The first component, having a period of ≈29 minutes (long ISO), was present both in the iris and eyeball movements. The second component, having a period of ≈2 minutes (short ISO), was observed only in the rhythmic constrictions and dilations of the pupils. Detected infra-slow oscillations (ISO) were present bilaterally, i.e., in both eyes simultaneously, and were synchronised between the left and right eyes. Moreover, the long infra-slow oscillation component was synchronised with the cyclic alternations of the brain state revealed by rhythmic changes in the pattern of the electrocorticographic (ECoG) signal.

### Technical Considerations

The possibility that the rhythmic changes in pupil size observed in our study are the result of rhythmic changes in external light intensity, which activates the light response reflex, should be considered. During the recording sessions, the eyes of the animals were exposed to two types of lighting. One source was the coaxial illumination of the video recording optic system; one source was used for each eye, and each source emitted constant, high-intensity, white light (see the Materials and Methods section) aimed at saturating all photoreceptors contributing to the encoding of irradiance [30]. Thus, this illumination should elicit maximal constriction of the animals’ pupils. The second source of illumination reaching the animals’ eyes consisted of light pulses (100 ms long) emitted every 100 s by the light emitting diodes (type: micro SMD) located near the temporal corner of each eye (see photographs in Fig. 1 and 6 in the Results section). The LEDs were emitting red (650±28 nm), low-intensity light, which causes an increase in irradiance that should be negligible and not perceived by already saturated photoreceptors. This assumption was confirmed by our recordings that show the lack of visually evoked cortical potentials driven by LED light pulses (data not shown). The lack of influence of the LED light pulses on the studied phenomenon is further confirmed by the lack of a frequency component corresponding to the interpulse interval (100 s; 0.01 Hz) in the ISOs observed in the eyes. Thus, generation of the described infra-slow oscillations by external, rhythmic activation of the pupillary light reflex seems highly unlikely.

Another potential “nonbrain” source of the observed changes of pupil size could be rotation of the eyeball itself. Our study has shown that centres of the pupils of urethane-anaesthetised rats change their position in a cyclic way. The largest distance (≈2 mm) between the extreme positions was observed in the nasal-temporal direction. Assuming that the diameter of the adult rat eyeball is approximately 6.4 mm [31], it can be calculated that a 2-mm shift of the pupil centre results from an eyeball rotation of approximately 39°. Because of this rotation, the observed diameter of pupil projection on a video plane can differ from the real value by approximately 30%. During our recordings, the video cameras were positioned in such a way that the optic axes of the eyes and cameras were aligned during cortical deactivation, that is, when the eyeballs were maximally rotated in the temporal direction. Successive phases of ECoG activation and eyeball rotation in the nasal direction should cause the observed area of the pupil to be smaller than the real one by approximately ≈34%. Nevertheless, in our study, we observed that the pupil areas (minimal and maximal) were significantly larger during activation than during cortical deactivation when the optical axes of the eyes and cameras were in line. This finding suggests that the observed, long infra-slow changes of the pupil area are a real phenomenon, not just an optical illusion.

### Possible Mechanism of the Observed Phenomenon

**Figure 8:**
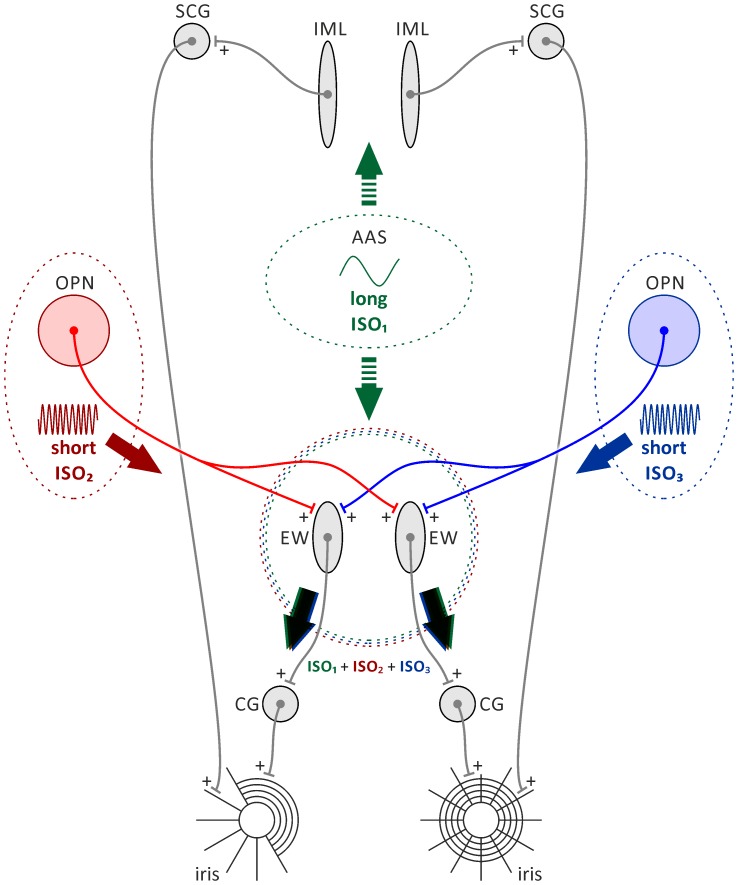
Diagram showing the functional model of the generation of infra-slow oscillations in the pupil size of urethane-anaesthetised rats. A detailed description of the model is given in the Results section. Note that green dashed arrows indicate plausibly polysynaptic route. Abbreviations: AAS, ascending activating system; OPN, olivary pretectal nucleus; IML, intermediolateral cell column; SCG, superior cervical ganglion; EW, Edinger-Westphal nucleus; CG, ciliary ganglion; ISO, infra-slow oscillation.

As mentioned in the Introduction, there are a variety of infra-slow oscillations that can be detected in mammalian brains (for a review, see [2]). It has been hypothesised that at least two known infra-slow brain rhythms, i.e. ISO at the level of neuronal firing in a few nuclei of the subcortical visual system and cyclic, sleep-like alternations in the brain state of urethane-anaesthetised rats, should be reflected by changes of the pupil area. Our observations confirm this hypothesis. As the basis for further, detailed studies, we propose a functional model of documented pupillary ISO generation (Fig. 8). Our model postulates that the observed rhythmic changes of pupil size result from an interference process of a few basic infra-slow oscillations that occur in the defined brain network. One of the basic oscillations is the cyclic change in the activity of the ascending activating system (AAS) that control brain state and balance in autonomic nervous system activity, and thus can influence the pupil size. As mentioned in the Introduction, sleep-like cyclic cortical activation observed under urethane anaesthesia depends upon activity of cholinergic nuclei of the basal forebrain and brain stem [22] – elements of the ascending activating system that control structure of natural sleep [32], [33]. High activity of AAS cholinergic neurons, inducing cortical activation, should result in the increase of the pupil size (iris dilations), as has been shown by Liu and Dacus [24]. Another group of brain oscillations that can influence the size of the pupil are infra-slow, rhythmic changes in the level of firing observed in a few nuclei of the subcortical visual system [8], [11], [21]. These oscillations are characterised by a period of ≈2 minutes and are synchronised within the brain hemisphere, i.e., there is zero phase shift between the ISO in ipsilateral structures. Additionally, there is no synchronisation between the hemispheres, i.e., there is no constant phase shift between the contralateral structures. Olivary pretectal nuclei (OPN) are key retinorecipient elements of the neuronal network subserving the pupillary light reflex [34], [35] and bilaterally innervate the Edinger-Westphal nuclei [36], [37]. According to our model, each EW nucleus receives oscillatory, excitatory input from bilaterally located OPNs (short ISO_2_ and short ISO_3_; Fig. 8) and modulatory input from the ascending activating system (long ISO_1_, Fig. 8), reflecting cyclic brain-state alternations. These oscillatory inputs are integrated (interfere) at the level of EW, and the resultant signal is conveyed to the parasympathetic neurons of the ipsilateral ciliary ganglion (CG) and further to the iris sphincter muscle, whose excitation constricts the pupil (Fig. 8).

Thus, the iris musculature receives two oscillatory signals, the first from the sympathetic branch and the second from the parasympathetic branch of the autonomic nervous system; we propose that these signals cause the rhythmic changes in the diameters of the pupils of anaesthetised rats, as observed in our study. During the experiments, we also observed cyclic, slow movements of the eyeballs. This phenomenon was correlated with the brain state and caused changes in the relative positions of the pupil centres, from the nasal position during cortical activation to the temporal position during cortical deactivation. Additionally, during the activation phase, we observed sporadic eye blinks and jaw movements of the animal. It has been shown that nuclei of the oculomotor complex receive cholinergic innervation [38]–[40] and that muscarinic receptors modulate activity of motoneurons in oculomotor nucleus [41], [42]. Moreover, the motoneurons of the facial nucleus [43] and trigeminal nucleus [44], [45] of the mammalian brain receive cholinergic innervation. Thus, increased activity of the ascending activating system during cortical activation could facilitate the eyeball movements, as well as occasional eye blinks and jaw movements of urethane-anaesthetised rats.

The phenomenon described in this study and the proposed model suggest that the cortical activation observed during urethane anaesthesia is associated with increased firing of the cholinergic neurons of ascending activating system of the brain, as has been suggested by Clement et. al [22]. To further test this hypothesis, it is necessary to conduct experiments during which the activity of the cholinergic neurons and cortical EEG is recorded over a few cycles of brain-state alternations in urethane-anaesthetised animals. Additionally, bilateral recording of the rhythmic neuronal activity from olivary pretectal nuclei would be necessary to verify the hypothesis stated here concerning the mechanism of generation of infra-slow oscillations in pupil size. With these three, presumably oscillatory signals, it should be possible to create a mathematical model of pupil size changes that considers the postulated interference of brain oscillations at the level of the Edinger-Westphal nucleus (ISO_1_+ ISO_2_+ ISO_3_) and the musculature of the iris (Fig. 8).

### Function and Significance

An open question is whether the observed rhythmic movements of the iris are merely a reflection of the brain oscillations or whether they have a physiological function. To date, there are no data with which to answer this question. A simple, direct benefit from iris movements could be stimulation of the blood circulation within the iris itself and/or facilitation of the aqueous humour flow from the posterior to anterior chamber of the eye. In fact, it has been shown that pupil dilation is accompanied by iris thickening and closure of the eye’s angle, which causes an obstruction in aqueous humour flow and a fast increase in intraocular pressure that can lead to acute angle-closure glaucoma [46]–[49]. Internally generated rhythmic constrictions of the pupils would prevent the intraocular pressure increase in nocturnal animals that are active for a long time periods in darkness by inducing pupil dilation. It has also been shown that small-diameter eyeballs and farsightedness causing a narrow angle of the eye are risk factors for glaucoma [50]. Thus, it seems possible that small nocturnal rodents would strongly benefit from such a mechanism.

The very fact that the result of interference of different brain infra-slow oscillations can be translated to rhythmic changes in the physiological parameter (i.e., pupil size) raises the possibility of similar control and/or influence on other physiological processes. This possibility seems especially plausible because extensive, often bilateral projections of the subcortical visual system nuclei displaying infra-slow oscillations have been described (i.e., IGL and OPN; [51], [11]). Such a broad network of IGL and OPN interconnections gives an anatomical basis for the integration of infra-slow oscillations generated by these nuclei by other brain networks involved in a broad spectrum of functions, including the control of oculomotor, vestibular and neuroendocrine systems and the regulation of sleep and/or circadian rhythms [52]-[56]. Additionally, our observations suggest a few straightforward factors that should be considered during the planning of further research and the interpretation of results obtained in past and future studies. Measured values of parameters describing physiological phenomena and/or behaviour of animals can depend on the phase of the infra-slow rhythms displayed in various brain networks. Thus, a time schedule of the recordings/observations should be planned to ensure coverage of all phases of potentially involved infra-slow rhythms to avoid obtaining incomplete pictures of the studied processes. Moreover, the very occurrence of some phenomena can be restricted to a certain phase of the particular brain infra-slow oscillation, and high variation of averaged parameters in some cases can result from rhythmic changes rather than from random instability.

In sum, our results indicate that different brain rhythms can be subjected to interference within defined brain networks, leading to the emergence of new, complex oscillations underlying changes in the physiology and behaviour of animals. Further research confirming and documenting the suggested mechanism of interference of oscillations within brain networks will establish a new scope for interpreting and understanding potential sources of variability in brain processes and brain-controlled functions.
